# Tuberculosis in Canada: Detection, Intervention and Compliance

**DOI:** 10.3934/publichealth.2014.4.241

**Published:** 2014-11-25

**Authors:** Katya Richardson, Beate Sander, Hongbin Guo, Amy Greer, Jane Heffernan

**Affiliations:** 1 Centre for Disease Modelling, York Institute for Health Research, York University, Toronto, Canada; 2 Public Health Ontario, Toronto, Canada; 3 Institute of Health Policy, Management and Evaluation, University of Toronto, Canada; 4 Institute for Clinical Evaluative Sciences, University of Toronto, Canada; 5 Centre for Communicable Diseases and Infection Control, Public Health Agency of Canada, Ottawa, Canada; 6 Department of Population Medicine, Ontario Veterinary College, University of Guelph, Guelph, Canada; 7 Department of Mathematics and Statistics, York University, Toronto, Canada

**Keywords:** tuberculosis, Canada, public health, detection, intervention, treatment, compliance, affected populations, mathematical modelling

## Abstract

This paper provides an overview of the current state of TB in Canada by referencing information presented at the workshop, “Tuberculosis: Detection, Prevention, and Compliance.” The workshop took place on November 14 and 15, 2012 in Ottawa. The workshop was organized by the Centre for Disease Modeling and the Public Health Agency of Canada as a two-day knowledge translation event that was comprised of scientific and policy focused presentations designed to address four key objectives: (1) Evaluate the success of current tuberculosis (TB) health policies and control strategies in Canada and for specific Canadian sub-populations; (2) Determine the impact of detection, intervention, compliance, and education strategies in terms of TB incidence and prevalence; (3) Develop targets for future interventions by identifying key characteristics of TB epidemics that impact the success of TB health policies and control strategies; (4) Leverage our existing ties with public health decision makers, aboriginal health organizations, and organizations serving the homeless to develop a research community that is based on close collaboration, and will foster national TB control efforts. The workshop elicited robust discussions between experts from a variety of academic disciplines and government officials. A summary of the information presented, comments shared, and questions posed, will provide a comprehensive understanding of the status of TB in Canada and future directions to be taken for improved control of the disease.

## 1. Introduction

Tuberculosis (TB) was once considered one of the deadliest infectious diseases in the world. In the early 19th century it was responsible for nearly one quarter of deaths in Europe [1]. Between the 1940s and 1970s, these rates declined significantly as a result of increased public awareness, improved living conditions, and the introduction of effective antibiotics [2]. Still, the disease plagues an average of 8.7 million globally with the most endemic regions being those in developing countries, where public health capacity to manage the disease is limited [3].

Due in part to the excellent health care and public health system in Canada, the country reports one of the lowest tuberculosis disease rates in the world; the estimated burden of disease was reported to be 5 per 100,000 population on average over the period of 2008–2010 [4]. However, in certain segments of the population, disease rates are much higher, most notably among foreign-born Canadians, indigenous populations (First Nations, Inuit, and Metis), and homeless populations in large cities. The disproportionate risk in these population groups has prompted the Federal Government to make tuberculosis control a public health priority. In 2006, Canada aligned itself with the United Nations Global Plan to Stop TB and set a goal to halve 1990 tuberculosis prevalence rates by 2015, reducing the national incidence rate to 3.6 cases per 100,000 population [5]. The Communicable and Infectious Disease Steering Committee is an arm of the Pan-Canadian Public Health Network, which reports to Canada's Deputy Ministers. It has launched a TB Task Group to increase Federal-Provincial-Territorial collaboration around the issue [6],[7]. To function, it must identify evidence-based, practical approaches for effective prevention, diagnosis, and treatment of TB in the key populations at risk.

The Centre for Disease Modelling (CDM), a research centre at York University, contributes research products to help inform evidence-based policies. The CDM operates at a national level to study, by way of mathematical modelling, the characteristics of disease transmission and the effectiveness and cost-effectiveness of various detection and treatment strategies [8]. Recognizing tuberculosis as a Canadian health priority, the CDM co-organized a two-day workshop with the Public Health Agency of Canada on the topic, entitled “Tuberculosis in Canada: Detection, Prevention, and Compliance”, which was held on November 14 and 15, 2012 in Ottawa [9]. This workshop acted as an important avenue for knowledge translation and the application of previous work on tuberculosis infection control in Canada. It also provided a national forum to discuss and formalize priorities for research activities and strengthen collaborative efforts. The primary objectives were to present recommendations on the detection, vaccination and treatment of TB, as well as education and compliance strategies. In addition, it sought to analyze the effects of TB and health policies in affected communities and their associated costs; and the role of mathematical modelling in advancing TB control in Canada. The organizers invited professionals from diverse fields in efforts to lead robust and informed discussions that would give way to practical solutions and concrete action plans. As such, the workshop involved Canadian public health professionals and planners; representatives for those populations most affected by this disease; and researchers in public health and medicine, social science, and infectious disease modelling.

## 2. Overview of Tuberculosis

Tuberculosis is an infectious disease caused by the bacterium *Mycobacterium tuberculosis* (*M. tuberculosis*). The disease usually affects the lungs, but it can also affect other parts of the body, including the kidneys, spine, and brain. TB can be spread from person to person when an infectious individual (with active tuberculosis) expels (i.e. coughs, sneezes, or even sings) droplet nuclei containing *M. tuberculosis* into the air [10],[11]. Transmission is most common between people who spend a lot of time within a confined indoor space. When an individual is exposed to the bacteria in the air (i.e. breathes it in) the individual can clear the pathogen via the immune system or the individual can become infected [10],[11].

Infection begins when *M. tuberculosis* is inhaled through the air and engulfed by alveolar macrophages, a major component of the immune system in the lung. The macrophages then invade the subtending epithelial layer, activating the immune response. This is followed by the migration of macrophages and T-lymphocytes to the site of infection through blood vessels. These cells as well as other immune cells initiate the formation of granuloma which limit the spread of bacteria and isolate infected macrophages from the rest of the lung. The *M. tuberculosis* bacteria can grow through division in infected macrophages, as well as extracellularly.

The majority of infected individuals will not progress to active TB disease after infection, and will have latent tuberculosis infection (LTBI). LTBI occurs when TB bacteria persist in the host without signs of active disease, and can grow to cause active disease in the future, after the host immune system fails to control the pathogen (called reactivation). Overall, without treatment, approximately 5–10% of infected persons will develop TB disease at some time in their lives, with about half of those developing active disease within the first two years of infection [10],[11]. The risk of developing TB is considerably higher in individuals with weak immune systems, such as those with HIV, and individuals on immunosuppressive therapies [10],[11]. However, mechanisms of TB latency and activation are still poorly understood. More research is needed to identify laboratory markers to predict the risk of progression from LTBI to active TB disease.

Although tuberculosis can be treated with antibiotics, drug resistance has become a major public health problem globally. Drug resistance arises due to improper use of antibiotics in TB patients that have drug-susceptible TB strains [11]. This may be due to the administration of improper treatment regimens, and failure of patients to complete the full course of treatment. A patient who develops active disease with a drug-resistant TB strain can transmit this form of TB to other individuals. Multidrug resistant TB (MDR-TB) refers to strains that are resistant to the two most powerful anti-TB drugs, isoniazid and rifampicin [11]. In these cases, a second line drug may be required to cure the patient. Resistance to TB drug therapies has been observed in Canada. In 2010, 1.3% of samples tested were cases of MDR-TB [12]. MDR-TB in Canada is associated with importation from countries where drug resistance has emerged or lack of compliance to TB treatments [6].

### 2.1 Prevention: Bacillus Calmette-Guérin vaccine

Historically, the Bacillus Calmette-Guérin (BCG) vaccine has been provided in many Canadian jurisdictions. However, with the declining rate of TB in many settings and concerns about the risk-benefit ratio associated with a live, attenuated vaccine, the BCG is currently only recommended in some high-incidence communities [6],[13]–[15]. The current Canadian recommendation is for infants in high-incidence settings [6]. BCG may also be administered to travelers intending on extended stays in high TB incidence countries where BCG is routinely given [6].

### 2.2 Detection: screening strategies

There are several methods for detecting tuberculosis in infected individuals. These include the Mantoux tuberculin skin test (TST), the Interferon-Gamma Release Assays (IGRA) test, and laboratory diagnostic tests such as GeneXpert MTB/RIF. Each method has its own advantages and disadvantages. The discussions taking place between policy makers centre on which method is most accurate and cost-effective, and how external factors may influence efficacy.

The procedure for the Mantoux test is to inject a standard dose of 0.1 mL tuberculin purified protein derivative (PPD) into the subdermal of the forearm. Between 48–72 hours after the injection, the patient returns to the clinic to have the size of the induration/skin reaction measured. The method for diagnosing TB depends on the level of risk held by each patient. For example, it is recommended that an HIV-infected individual (high-risk) be diagnosed with TB if an induration of ≥ 5 mm is measured [16], but a person with no known risk factors for TB (low-risk) be diagnosed for an induration of ≥ 10 mm [16].

The IGRA test is a whole blood test, which measures an individual's immune reactivity to M. tuberculosis. The testing process requires collecting blood samples, which are incubated overnight with M. tuberculosis antigen, after which a measurement is taken for interferon-gamma (IFN-γ). In someone who has been previously exposed to TB, the M tuberculosis antigens will stimulate blood lymphocytes to produce gamma interferon [17]. Since both TST and IGRA depend on an intact immune system, both are likely to have lower sensitivities in immuno-compromised individuals and neither can distinguish between active TB disease and LTBI [17]. Still, IGRAs have advantages over TSTs such as higher specificity in patients with non-tuberculous mycobacterial infection and in patients who have been vaccinated with BCG, especially if BCG is given after infancy or multiple times [17]. Higher specificity of the IGRA test was observed in a study conducted in a correctional facility in Kingston, Ontario, which found that only 32% of prisoners who tested positive with a skin test also tested positive with an IGRA test [18]. Prisoners who had been vaccinated with BCG were also 3.2 times less likely to test positive with IGRA screening. As a result of increased specificity, treatment uptake was significantly reduced [18]. Furthermore, it is easier to obtain results from IGRA screening because the patient is not required to return to the clinic within 48–72 hours for TST reading, which can be problematic for some patients. Knowing that IGRAs reduce the probability of false-positives, the next important question is when TSTs or IGRAs or both should be used for a more cost-effective approach [17],[19]. This is an area where mathematical modellers can provide quantitative studies for policy analysis.

Traditional solid and liquid culture diagnostic systems, which are used to diagnose active TB disease, require weeks to grow and several more weeks before drug resistance can be determined. Within the last 10 years, a fully automated alternative has emerged: the Rapid MTB/RIF Xpert assay. This is a cartridge-based nucleic acid amplification test (NAAT) that simultaneously detects TB and rifampin resistance, which is a marker for multi-drug resistant strains. The GeneXpert, which is the best test to date, requires as little as two hours to produce results and detects 85% of smear-positive TB [17]. A study conducted in South Africa found that GeneXpert increased the number of TB cases diagnosed by 30–37% and the number of MDR-TB cases diagnosed by 69–71% [20]. Other advantages of the machine are that it requires minimal training and short hands-on time, while reducing the possibility of cross contamination. In 2010, the World Health Organization published a strong recommendation that GeneXpert be used as the initial diagnostic test in individuals suspected of having MDR-TB or HIV-associated TB, rather than traditional microscopy [17], [21]. Although this form of testing requires a high capital investment for machines and cartridges, mathematical models can be used to estimate potential cost savings. These savings are a result of earlier detection and treatment initiation which improves health outcomes and reduces TB transmission. In a model created by the Institute National de Santé Publique of Quebec, the cost of treatment if diagnosed by the GeneXpert, was reduced from $4,800 to $970 [20]. This is due to the fact that traditional laboratory diagnostic tools require approximately 12 days to produce results, during which time it was assumed that a patient suspected of carrying active pulmonary TB is hospitalized in isolation. The analysis will vary if home isolation is also considered. GeneXpert may be more cost-effective in high-incidence regions and remote communities. Studies are currently being conducted in Quebec to determine its cost-effectiveness in northern regions of the province where incidence rates are high and hospital isolation rooms are few [20].

In addition to the methods employed for detecting tuberculosis in individuals, scientists have recently begun sequencing whole genomes, which in combination with field epidemiology; can be used to establish networks of transmission in populations. During a tuberculosis outbreak, isolates can be genotyped and compared to those of other TB-positive individuals. Identical restriction fragment length polymorphism (RFLP), spoligotype, or mycobacterial interspersed repetitive unit variable number tandem repeat (MIRU-VNTR) patterns suggest that cases belong to a cluster [22]. Now, with next-generation sequencers making it easier to process whole genomes, scientists are able to look for gene mutations which can act as markers for transmission between individuals. Along with sequencing, contact network analysis can be conducted to map out transmission networks. This involves interviewing patients and generating a list of all individuals who have come into close contact with the patient. This is an exciting new field that has been successful in establishing the source of transmission in a number of outbreaks in Canada and internationally [23]. However, it does come with challenges, which are often due to the complex nature of the disease. For example, it can be difficult to determine the genomic sequence of a “super spreader” whose infection has most likely mutated from the time of infection to the time of separate transmissions [22]. Furthermore, it is unknown how mutation rates differ between LTBI and active TB. Despite the vigorous work being conducted by provincial health agencies and laboratories in field epidemiology, it is uncertain whether resources are effectively deployed to complete the laborious task of establishing contact networks. In British Columbia only 20% of isolates are genotyped because it is only performed on the recommendation of health practitioners who suspect recent transmission [22]. Nevertheless, with the estimated cost of sequencing a genome at < $60, it is important to consider whether this is a viable alternative to the GeneXpert, which requires cartridges priced at $60 each. This is undoubtedly a highly advanced methodology for TB detection, but when modeling its potential benefits, it needs to be put into context. While genotyping may not be feasible for remote and isolated communities, it may be the fastest way to prevent the spread of an outbreak in a large urban centre where lab results only require a few days and field epidemiologists can track contacts with infectious individuals.

### 2.3 Treatment and compliance

The recommended treatments for both LTBI and TB disease are the prescription of antibiotic regimens. Patients with LTBI may be prescribed isoniazid (INH) or rifampin (RMP) for a course of 6–9 months, while patients who have active TB disease may be prescribed a combination or three or more drugs including isoniazid, rifampin, ethambutol (EMB), pyrazinamide (PZA) [10]. Antibiotic treatment can prevent LTBI from developing into active disease. Within a few weeks of starting appropriate treatments, most patients with TB disease will no longer be contagious but it is necessary to complete the full course of treatment. Failing to complete treatment can lead to drug-resistance, and it can cause a patient to become infectious again and symptoms could worsen.

Despite the serious consequences of failing to complete treatment, compliance rates are generally not sufficient. The most likely reason for low compliance is the long course of treatment. Directly observed therapy (DOT) programs have been successful in increasing TB treatment compliance, and video DOT (VDOT) programs have been even more successful in areas/conditions where social and geographic barriers have existed [23],[24]. However, DOT and VDOT still experience barriers and costs that could be reduced if the course of TB treatment were shortened. Studies on shortened TB treatment efficacies have been conducted recently. The CDC has reported that results from three randomized controlled trials show that a 12 week combined treatment of isoniazid-rifapentine could cure the disease, increase compliance rates, and reduce side effects. In these studies 82% of the people on the 3-month regimen completed the full treatment, while only 69% completed the 9-month regimen [25]. In another recent study published by Sterling, Villarino, et al., it was concluded that a combined 3-month treatment of rifampentine and isoniazid was at least as effective as a 9-month treatment of isoniazid for LTBI [26]. Currently, rifapentine is not commercially available in Canada. However, if approved it would be important to determine the cost-effectiveness of shortening treatments. The 3-month regimen of rifapentine is more expensive, costing about $155, while the 9-month course of isoniazid costs less than $6 for the period [26].

After effective short-course therapy for active TB, it is possible that some individuals may experience another recurrent TB episode. The recurrent episode may be due to endogenous reactivation or exogenous reinfection. Whether the development of active tuberculosis in people with previous tuberculosis infection represents an episode of endogenous reactivation or exogenous reinfection has been debated for decades. However, with the new molecular techniques available for detection, it has become possible to determine whether a case of recurrent TB has developed from reactivation or exogenous reinfection. Recently acquired infections will show clusters, while reactivated cases will show unique sequences, which are results of the geographical distance from the source of infection and the activation, and length of time between infection and activation. In Canada, the majority of active TB disease cases are a result of reactivation of TB in LTBI patients [6]. In Toronto, every year there are approximately 2000 reported contacts of active TB and about 1400 medical surveillance referrals for newly landed migrants [27]. Approximately 70 staff are employed in the TB Program in Toronto to conduct contact follow-up for all TB cases to identify if there has been any transmission. Small chains of TB transmission involving 2–5 individuals have been reported [27].

### 2.4 Newcomers

Although foreign-born individuals only represented 22% of the Canadian population in 2010, they constituted 66% of all reported tuberculosis cases in Canada in that year [28]. It is believed that this disproportionate epidemiology is primarily driven by the reactivation of LTBI post-landing and increased immigration from high incidence countries [6],[28]. The Immigration Medical Examination (IME), which is required for all individuals applying for permanent residency and certain individuals applying for temporary residency in Canada, incorporates TB testing in an effort to detect prevalent active TB in migrants and ensure that they are treated and no longer infectious upon arrival [6]. An effective strategy for preventing inactive TB from becoming active is to perform regular post-landing follow-ups and ongoing monitoring of the condition. Those with evidence of pulmonary TB infection, including a history of active TB, and abnormal chest x-rays suggestive of TB infection/disease, require post-landing medical surveillance. These migrants are instructed to contact the provincial health authority within 30 days of landing. Post-landing surveillance can span between 0 years (patient does not contact the health authority) to 10 years, however the majority of cases are monitored for two years [19].

Research has been conducted to determine whether current IME guidelines are effective in detecting and treating active TB in migrant populations. In a study conducted by the BCCDC, an incidence rate of 42.2 per 100,000 entries between 2004 and 2010 was observed, where landed immigrants and humanitarian populations had the highest incidence rates of 66.9 and 49.3 respectively [19]. Within the landed immigrants cohort, the study found that 75% of cases were activated within the first 3 years of arrival [19]. Overall, 35% (107/308) of active cases found were in people referred for post landing surveillance, suggesting that policies that measure and improve adherence to IME post-landing surveillance may be a “quick win” to improve TB screening [19]. Current IME screening appears to be effective for recent migrants, but may not be effective for those in Canada > 5 years (representing a significant fraction of the immigrant cohort). A collaboration between Citizenship and Immigration Canada (CIC) and provincial agencies could be undertaken to identify “late” cases of active TB in the foreign born population however [19]. The study also found that groups with particularly high incidence are predicted by the TB incidence in their country of birth (particularly those larger than 100/100,000). The highest incidence countries were identified as the Philippines, India, China, and Vietnam [19]. A recent set of screening strategies published by Pottie et al. in 2011 in the *Canadian Medical Association Journal* (CMAJ) recommended that 1) all refugees aged 20–50 years be screened and 2) all other migrants with risk factors for TB be screened (as per TB Standards [6]); [29]. However, there is meaningful variation in TB incidence by immigration classification, country of birth, and time since arrival to suggest that screening strategies can be more targeted in accordance with the collected data.

A modelling study was conducted at the University of Alberta to determine how screening strategies for migrants could be better targeted to decrease TB incidence to help meet Canada's Stop TB goal. The procedure of performing skin tests followed by IGRAs and treating LTBI with a 9-month course of INH, was held constant in the study. The study incorporated a pooled sensitivity and specificity of 80% and 75%, respectively for the tuberculin skin test, and a pooled sensitivity and specificity of 83% and 97%, respectively for the IGRAs. The model indicated that if 100% of migrants from high-incidence countries (incidence rate > 50 cases/100,000 population) and 100% of migrants from medium-incidence countries (incidence rate 15–50 cases/100,000 population) were screened and treated for LTBI, Canada would meet its goal of reducing incidence rates to 3.6 per 100,000 population by 2015 [30]. However, it was also found that this goal could only be met if 100% of the high-incidence group were screened [30]. An even more targeted approach would be to screen all migrants from high-incidence countries younger than 35 years. In this case, Canada would not meet its goal, but study results show that a downward trend could be observed and the incidence rate would fall below 3.6 per 100,000 population in a few more years [30].

### 2.5 Aboriginal populations in Canada

Tuberculosis disproportionately affects Canada's Aboriginal populations (Inuit, Métis, and First Nations). While constituting only 4% of the Canadian population, Aboriginal populations represented 21% of all tuberculosis cases in Canada in 2010 [28]. There is some variation in TB incidence rates between rural and urban areas, and by Aboriginal populations. For instance, although incidence rates have decreased over the period of 1994–2008 for First Nations and Métis populations, it has increased drastically for the Inuit population [28]. This suggests that we need a targeted approach for addressing TB. Furthermore, it has been observed that young adults are now the age group most affected by TB in Inuit populations, and that many of those infected do not present the classic TB symptoms [28],[31]. Thus, cases could quite easily be under-diagnosed. Efforts are underway to reduce incidence rates, but tackling tuberculosis within Aboriginal communities poses a major public health challenge.

First, some hospitals and systems of care in Inuit communities lack some of the necessary medical equipment for detection of tuberculosis. For some remote locations, sputum samples collected for analysis must be shipped by air to laboratories in large cities for diagnostic testing and results could take several weeks to be communicated to the patient, effectively prolonging exposure time for contacts [31]. In remote and northern communities, travel can be required to receive secondary and tertiary care, which often means days or weeks away from family and social support, as well as the added cost of accommodation and meals. In addition to lack of equipment, communities struggle to attract and keep well-trained nurses, doctors and other health care providers [31]–[33].

A second challenge resides in the negative feelings and distrust expressed by individuals in Aboriginal populations with respect to past tuberculosis public health practices/policies. In some cases, public health practices required infected individuals to be transported to hospitals and sanatoria to be treated for tuberculosis, but limited information was provided to their families. Infected individuals sometimes did not return to their communities [31],[34],[35]. Thus, the requirement to travel for treatment or follow-up is further affected by this historical stigma.

Additionally, it is vital to address the significant health disparities experienced by Aboriginal populations in Canada as part of a broader framework that incorporates social determinants of health. Important contributors to disease susceptibility within Aboriginal communities include crowded and inadequately ventilated housing; malnutrition; substance dependence and/or cigarette smoking; and diabetes. Tackling these social and health inequalities could have an impact on reducing the risk for TB [31].

Standard and best practices for addressing TB are outlined in the Canadian TB Standards [6], and in “Health Canada's Strategy against Tuberculosis for First Nations On-Reserve” [36]. The strategy is aligned with national tuberculosis guidance documents [6],[7]. It focuses on: preventing, diagnosing and managing TB; targeting populations at greatest risk for TB; developing and maintaining partnerships; and continuous quality improvement [36],[37]. The strategy recognizes that partnerships are essential for the seamless delivery of care and for addressing cross-cutting issues such as the social determinants of health. As such, Health Canada works closely with First Nations leadership and communities, provincial programs, regional/local health authorities, TB experts and the Public Health Agency of Canada to either provide TB services or assure they are accessible to First Nations living on-reserve [36],[37].

Improving access to health services in First Nations and Inuit communities could address inequalities and in turn, reduce rates of TB. For example, diagnostic technologies such as the GeneXpert which are automated and require little hands-on operation and training could be provided to communities that lack trained professionals [e.g.,31]. Also, chest x-ray machines could be provided to communities for follow-up so that travel is not required [31].

Recently, the TAIMA TB project, co-funded by PHAC, CIHR and Nunavut and led by researchers from the Ottawa Hospital Research Institute and the University of Ottawa, has made important advances in TB education and testing in Nunavut. The success of the TAIMA TB project is largely attributed to its grassroots level operation. The project aims to raise TB awareness by way of community empowerment. The TAIMA TB project is guided by a steering committee including representatives from Nunavut Tunngavik and the Government of Nunavut, and works closely with community members at every stage of the project to decide on best methods for sharing information on TB, encouraging testing, and the rollout of in-home testing procedures [38]. Within this project, education programs were successful in relating and informing community members about the disease, and in-home testing had a large participation rate [38]. This project has also seen the first feasible use of IGRA in Nunavut, and has been extended for the introduction of GeneXpert in a Nunavut hospital [38].

### 2.6 Homelessness and TB

The homeless populations in Canada's large cities also incur higher rates of TB incidence. A meta-analysis of 43 studies found the incidence rate of TB infection in homeless populations to range from 0.2% to 7.7% [39]. In 2001, a serious outbreak of TB in the emergency shelters of Toronto led to 15 active TB cases and three related deaths. Following a coroner's inquest, control guidelines for shelters and drop-in centres in Toronto were published. This publication represented an important step in the development of a coherent strategy for emergency response, but TB control does not simply involve management of the problem, it also involves addressing underlying causes. For example, social determinants of health account for 60–90% of many illnesses, including TB [40] and need to be examined in order to understand the higher incidence of TB infection among certain segments of the population. The homeless population is especially susceptible to TB infection because they experience malnutrition, suffer from mental health disorders and addictions, and have predisposing medical conditions such as Hepatitis C, HIV, chronic pulmonary disease, and untreated diabetes which can cause weakened immune systems.

In Toronto, on any given night there are over 5,000 people who are homeless [39]. The city's institutional responses to homelessness tend to focus on emergency responses, which have several drawbacks in terms of preventing TB transmission. Some aspects of this approach have included forced movements on the homeless which limits control over whom they are in contact with and often leads to interactions with other at-risk groups such as new migrants, Aboriginals, and prisoners. It also dictates that most homeless individuals' activities take place within congregate living conditions such as busy shelters, which are optimal environments for TB transmission. A study produced by the Canadian Homelessness Research Network (CHRN) concluded that emergency responses to homelessness are extremely expensive due to the high costs of providing beds in shelters, as well as the externalities caused by health problems and criminal engagement [39]. By focusing on structural changes, the City would approach TB from a preventative approach, which would be expected to improve health outcomes and reduce costs. For instance, the CHRN found that rent supplements and social housing, which provide better living conditions and reduce the opportunity for TB transmission, are viable alternatives to shelters [39].

Attempts have been made to study TB transmission within homeless populations but it is very difficult to trace contact networks once a case of active TB has been identified. This is due primarily to the chaotic lifestyle of the homeless, which involves interaction with other highly susceptible risk groups and a great deal of variability in status as homeless. A study which analyzed patterns of emergency shelter stays, found that 88% of the homeless population is only temporarily homeless, meaning they are homeless for less than a month, while 10% are episodic and 2% are chronic [39]. This means that the homeless population is not a discrete population. A homeless individual may move in and out of homelessness. Despite these challenges, a mathematical modeling research team at York University developed an epidemiological model to generate a better understanding of TB transmission among the homeless population in Canada. Results from this study can be used to identify improved strategies for detection and treatment, as well as how to avoid TB outbreaks in the future. Results showed that without external latent cases entering the homeless population from other TB affected populations (a primary simplistic assumption), it is possible to eliminate TB in the homeless population [41]. More work is to be done, employing more realistic assumptions (Table 1).

**Table 1.** Research opportunities.

Population	Study/Goals	Research methods/strategy
Aboriginal	1) Economic evaluation of increased screening including skin test and IGRA	Modelling, simulation, cost-effectiveness analysis, surveillance, community engagement
	2) Economic evaluation of improving the social determinants of health (i.e., housing)	
	3) Treatment with better compliance rates as a result of DOT and VDOT programs, as well as shortened treatment regiments	
	4) Comparisons between 1–3 above	
Foreign-born	1) Economic evaluation of increased screening including of immigrants from countries with high TB prevalence	Modelling and simulation, cost-effectiveness analysis, surveillance
	2) Economic evaluation of eliminating the screening of immigrants from countries with low TB prevalence	
	3) Provide specific definitions of “high” and “low” for 1–2 above	
	4) Follow-up periods needed for immigrants coming from countries with different TB prevalence levels to ensure TB activations will be recognized early	
All TB afflicted populations	1) Study interconnections between Aboriginal, foreign-born, homeless and correctional institute populations	Surveillance, modelling, simulation, laboratory
	2) Identify healthcare strategies that can reduce transmission and aid in genotyping studies when active disease is reported (contact tracing)	
Cell and pathogen load in-host	1) Identify key characteristics of TB infection in-host which indicate LTBI infection and activation	Modeling, simulation, laboratory
	2) Develop models that can be used to study the evolution of drug resistance in-host, and treatment strategies for drug-resistant infected TB patients	

### 2.7 TB infection in-host

To effectively treat and detect TB in patients, several components of the disease must be better understood. These include the mechanisms allowing for LTBI and disease activation, as well as the evolution of drug resistance. Yet, in-host data on TB infection is difficult to attain, making studies of in-host TB progression difficult to conduct. This is an area where mathematical models, can be effective [42]–[45]. A mathematical modeling research team at York University has developed a model of TB pathogen interaction with the immune system to determine key factors of in-host disease progression. This can predict disease clearance, LTBI, and progression to active disease. This study is ongoing. This work will also be extended to study the evolution of drug resistance, and the factors that influence TB transmission.

## 3. Discussion

In the discussion periods of the workshop, participants identified gaps in knowledge where further research is needed so that health care discrepancies can be addressed, and so that individual care strategies may be developed. These recommendations are summarized in Table 1. Interaction between public health officials and mathematical modelers, gave way to the identification of areas where modeling, specifically, would be an asset. For example, modeling could be used to develop economic evaluations of different treatment and screening strategies by target population, and for individuals with different disease progression experiences. There was also discussion about enhancing the integration of epidemiological data with mathematical models to provide better insight into important public health questions. It was suggested that social ties and networks of interactions be included in a TB modeling framework, as this would allow an in-depth study on changes in TB incidence with respect to patterns of social interactions.

During the discussion periods participants also discussed the current TB health policies in terms of detection and monitoring of latent and active cases, including any delays in TB diagnosis and TB diagnosis in remote communities. Participants shared knowledge of medical equipment allocations, contact tracing procedures, and monitoring policies of high-risk groups. Research questions were identified through the discussion session.

Recognizing that TB incidence is highly dependent on socio-economic conditions, participants explored the topic of social determinants of health with relation to TB affected populations. In doing so, it was agreed that, despite past research efforts, results could be enhanced by conducting further research into determinants and causal paths to TB. These may include housing, food security, mental wellness and access to health services. Results could also be enhanced through an analysis of TB co-morbidities, such as nutrition studies, and other cultural or social practices that affect the general well-being of a population (i.e., smoking, substance abuse).

A number of workshop presentations highlighted some effective TB education programs. Discussion centred on the use of different media facets (i.e., flyers, webpage, twitter) available, and the measurements that can be used to determine their effectiveness in encouraging the public to seek diagnosis and follow treatment plans. The STOP TB CANADA newsletter, which was recently re-launched, was noted for bringing together academics, government, NGOs and community engagement groups.

Finally, workshop participants discussed strategies for building partnerships between TB researchers, public health decisions makers, and community representatives. It was agreed that community engagement and empowerment are critical considerations. Participants proposed that a successful Community of Practice can be achieved by developing shared meanings through social engagements and interactions. It is important that communication be kept clear and concise, with a focus on using a common language that is accessible for all. Effective communication is needed to understand where tensions lie and how they can be addressed. This will allow for Communities of Practice to flourish, and enable them to tackle difficult questions on TB.

## 4. Conclusion

TB incidence rates in Canada are among the lowest in the world; however, the burden of disease is concentrated in several Canadian subpopulations. This workshop brought together individuals whose work focuses on Canadian TB afflicted populations from both urban areas and remote and isolated northern communities. Participants included public health decision makers and planners; researchers in public health, medicine, social science, health economics, and infectious disease modeling; and experts in TB epidemiology and medicine. Presentations provided important perspective into the areas of public health, screening, treatment, the social determinants of health, and scientific research, including mathematical modeling. A wealth of knowledge and experiences were shared, and critical factors affecting TB incidence, prevalence, and the success of intervention strategies were highlighted. The discussion periods were particularly productive, as participants identified strategies for TB detection and prevention for each target population. A key outcome of the workshop was the establishment of a set of research goals and methods for collaboration. The participation of diverse researchers and stakeholders at the current TB workshop attests to the successes of the CDM-PHAC collaborative initiatives. The participants agreed, in principle, to a national collaboration effort on TB in Canada. Within the CDM-PHAC collaboration this modeling network will move forward to achieve the identified goals, and ultimately, change the TB affected landscape in Canada.
